# Surrogate modelling for the prediction of spatial fields based on simultaneous dimensionality reduction of high-dimensional input/output spaces

**DOI:** 10.1098/rsos.171933

**Published:** 2018-04-25

**Authors:** D. Crevillén-García

**Affiliations:** School of Engineering, University of Warwick, Coventry CV4 7AL, UK

**Keywords:** stochastic PDE, simultaneous dimensionality reduction, Gaussian process regression, spatial field emulation

## Abstract

Time-consuming numerical simulators for solving groundwater flow and dissolution models of physico-chemical processes in deep aquifers normally require some of the model inputs to be defined in high-dimensional spaces in order to return realistic results. Sometimes, the outputs of interest are spatial fields leading to high-dimensional output spaces. Although Gaussian process emulation has been satisfactorily used for computing faithful and inexpensive approximations of complex simulators, these have been mostly applied to problems defined in low-dimensional input spaces. In this paper, we propose a method for simultaneously reducing the dimensionality of very high-dimensional input and output spaces in Gaussian process emulators for stochastic partial differential equation models while retaining the qualitative features of the original models. This allows us to build a surrogate model for the prediction of spatial fields in such time-consuming simulators. We apply the methodology to a model of convection and dissolution processes occurring during carbon capture and storage.

## 1. Introduction

The use of complex mathematical models for simulating and predicting the behaviour of physico-chemical processes is nowadays crucial in a broad range of groundwater disciplines, including contaminant transport and geological storage of CO_2_ in deep saline aquifers among many others. The complexity of these models normally involves the implementation of highly demanding and time-consuming numerical codes, and thus there is growing interest in designing faster and reliable statistical approximations of the computationally expensive simulators, so-called emulators.

The vast majority of physico-chemical processes in porous media can be successfully described using stochastic partial differential equations (SPDEs) (see [1–5]). One parameter of special relevance in these equations is the permeability of the porous medium used to describe the inherent random heterogeneity of the rock formation. Several researchers have shown in the past that although permeability values can exhibit large spatial variations, these variations are not entirely random but spatially correlated (e.g. [1–3]). It has been also shown through experimental validation that permeability fields can be successfully modelled using a log-Gaussian distribution assumption (e.g. [4]). For instance, Mara *et al.* [6] modelled strongly heterogeneous aquifers by using a stochastic Gaussian process (GP) for the log-transmissivity fields conditional on data sampled at a set of locations in an aquifer. One of the most extended possibilities to generate samples of a log-Gaussian random field is through Karhunen–Lo$\overset{´}{e}$ve (KL) decompositions of the correlation function of the underlying Gaussian field evaluated at each of the grid points of the computational domain (see [5,7]). These samples are represented by a sequence of indexed random coefficients in a finite series. An immediate consequence of this form of representation of the input fields is that the dimension of the stochastic input space is as high as the number of grid points in the computational domain. As an example, for standard meshes of size 50×50, 80×80 or 100×100 in a two-dimensional rectangular domain, we would need to deal with a few thousands of stochastic degrees of freedom (DoF) if we do not wish field variance preservation to become an issue. In other words, the hypothetical over-smoothing of the generated permeability fields caused by a further truncation of the original KL decomposition, if not handled properly, would lead to unrealistic results.

During the past decade, several high-dimensional model representation (HDMR) techniques, e.g. CUT-HDMR, adaptive HDMR and new truncated HDMR, have been developed to reduce the high-dimensionality of stochastic input spaces (see [8–12]). HDMR methods split the original high-dimensional model into a set of lower-dimensional sub-models leading to less computational effort when solving the resulting sub-models by any numerical method. One of the preferred methods to be complemented with HDMR techniques for solving high-dimensional SPDEs found in the literature is the stochastic collocation (SC) method in any of its variants, e.g. full-tensor product, Smolyak sparse grid etc. (see [11,13–17]). The SC method became so popular because, besides providing fast convergence, it lies within the so-called non-intrusive methods for solving SPDEs, i.e. neither knowledge nor algebraic manipulation of the equations that will be solved is required, and thus researchers can use existing in-house or commercial numerical simulators to implement the method. However, for some groundwater models (e.g. [5,7,18]) the number of stochastic DoF needed for an acceptable resolution of the results is still prohibitive for this method. One of the alternatives to overcome the problem of high dimensionality in time-demanding groundwater model simulators is GP emulation [19–21].

There are several applications of GP emulation of multivariate simulators, for instance Bowman *et al.* [22] compared four different techniques for emulating multivariate outputs in atmospheric dispersion models. To the best of our knowledge, there are still a limited number of publications in the literature dealing with GP emulation of groundwater models in very high-dimensional input spaces (e.g. [5]). GP emulators for high-dimensional simulators also necessitate HDMR methods to overcome the limitations of Bayesian regression. These limitations frequently arise in the estimation of some of the model parameters (so-called hyperparameters) present in anisotropic covariance/correlation functions. The hyperparameters are *a priori* unknown and need to be estimated from the data provided by the simulator. The maximum-likelihood estimate (MLE) method has been extensively employed to find estimates of the hyperparameters (see [5,23,24]). Most of the optimization algorithms used to find the MLE (in our case by minimizing the negative log marginal likelihood), for instance steepest descent, conjugate gradient, Hessian-free Newton etc., are critically dependent on the selection of the initial guess to initialize the iterative algorithm. This sensitivity to the choice of the initial values might be, for instance, due to the existence of multiple local maxima in the marginal likelihood [21]. While these methods have been satisfactorily used to estimate hyperparameters defined in low-dimensional spaces, for high-dimensional spaces this has historically led to an optimization problem, and in most of the cases to complete failure. In groundwater models, GP emulators normally represent point correlation by using automatic relevance determination covariances [25], and for these cases, we propose a continuation algorithm for passing the right initial values to the MLE method in the successive iterations. This will overcome the optimization issue and will make feasible the MLE method for a moderate/high dimension of the input space.

The focus of this paper is the development of a new approach for constructing GP emulators to act as a *full* surrogate model for computationally expensive *spatial field* simulators defined in very high-dimensional input and output spaces, and in particular for groundwater model simulators. Note that standard *scalar* GP emulation has already been successfully applied to complex and time-consuming scalar valued simulators [5]. Thus, the inputs and outputs of the simulator considered here will be spatial fields defined in very high-dimensional input and output spaces. The GP emulator is able to reproduce (up to a predetermined level of accuracy) the work of the computer model much faster. This is of vital importance in applications such as uncertainty quantification, design optimization and decision theory, where a large number (sometimes millions) of calls to the numerical simulator are required in order to produce a critical assessment. The methodology of the empirical simultaneous GP model reduction (ESGPMR) approach presented in this paper consists of combining two main techniques. (i) We use the method proposed by Higdon *et al.* [26] to reduce the dimensionality of the output space by using principal component analysis (PCA) and separate independent GP emulators for the coefficients in the PCA basis. Higdon’s approach has been successfully adapted to other applications, for example, Holden *et al.* [27] applied a variation of the method to high-dimensional climate model outputs. Bowman & Woods [22] adapted the method to the field of atmospheric dispersion by using the thin-plate splines technique. (ii) We capture the high-dimensional relationship between the simulator inputs and the coefficients of each of the vectors spanning the reduced output space by exploiting the properties of the KL decomposition of the input permeability fields and using cross-validation (CV). Thus, we find a subspace of the high-dimensional input space leading to an optimal representation of the GP model response surface. To test the GP emulation results, we take as reference (*true value*) a sample of 256 full numerical simulations. The simulations were obtained over 18 days of continuous intensive CPU computations on a 12-core Intel Xeon cluster processor. The time spent to compute the final prediction of the same 256 spatial fields with the ESGPMR approach on the same processor was 4 h.

The outline of this paper is as follows. In §2, we introduce the computationally expensive numerical simulator of a convection and dissolution process in random heterogeneous porous media. In §3, we describe the framework of the GP emulation methodology. We present the novel method for simultaneous input–output model dimension reduction and we detail how to properly estimate the hyperparameters of a high-dimensional space by using a continuation algorithm. In §4, we test the GP model reduction methodologies with the model problem introduced earlier. Concluding remarks are provided in §5.

## 2. Mathematical model and numerical simulator

Dissolution of CO_2_ in deep saline aquifers is considered one of the most effective ways of carbon capture and storage [28]. The model studied here focusses on the hydrodynamical part of the problem by setting a model for CO_2_-loaded flows in an idealized two-dimensional geometry. It considers the impact of hydrodynamic dispersion (or dispersivity), permeability heterogeneity and isotropy in porous media on the development of convecting instabilities. For solving the resulting problem, the finite-element method (FEM) is employed. The existence of continuous bifurcations from the no-flow steady-state solutions of the problem adds additional challenge to the search of numerical solutions, and to overcome this an arclength continuation technique [29] is used in conjunction with the FEM.

### 2.1. Convectively enhanced dissolution process in porous media

The dissolution of CO_2_ into the brine of the storage site causes an increase in the density of the mixture, leading the CO_2_ to sink while reacting with local rock minerals to become a solid carbonate [30]. This leads to the onset of convection rolls and the resulting mixing leads to a greater contact between the injected CO_2_ and local minerals, significantly enhancing the carbon capture. This process is known as convectively enhanced dissolution (C-ED) [31,32].

In recent theoretical and numerical works (e.g. [32–34]), researchers have investigated the behaviour of CO_2_ in deep saline aquifers. These studies focussed on the understanding of a simplified and idealized case where the problem is reduced to the motion of a fluid through a porous medium and where the dispersive transport is based on pure molecular diffusion. This paper will take into account more characteristics of natural aquifers, namely the rock heterogeneity and the hydrodynamic dispersion. This later will be modelled by a dispersion tensor, **D**, dependent on the local Darcy velocity of the fluid **u** as follows [34–36]: $\text{D}=D_{m}I+\beta_{T}∥\text{u}∥I+(\beta_{L}-\beta_{T})(\text{u}⊗\text{u}/∥\text{u}∥)$, where ⊗ represents the tensor product, I is the unit (identity) tensor, *D*_*m*_ is the molecular diffusion coefficient of the solute in the fluid and *β*_*L*_ and *β*_*T*_ are, respectively, the longitudinal and transverse dispersion coefficients, which satisfy *β*_*L*_≥*β*_*T*_≥0 [5,37,38].

We consider the C-ED process to occur in a two-dimensional domain representing a random, heterogeneous, isotropic porous medium of depth 2*H* and length *L*. The spatial variable is defined by **x**=(*x*,*z*) on the domain [0,*L*]×[−*H*,*H*]. The governing equations for this model are continuity (2.1), Darcy’s Law (2.2) and convection–diffusion-reaction (2.3) [32,33,38]:

2.1 $$\begin{array}{r} \nabla \cdot \text{u}=0, \end{array}$$

2.2 $$\begin{array}{r} \text{u}=-\frac{K}{\mu }(\nabla P+\rho g{\text{e}}_{z}) \end{array}$$

2.3 $$\begin{array}{rl} \;\mathrm{and}\;\phi \frac{\partial C}{\partial t}+\text{u}\cdot \nabla C & =\phi \nabla \cdot (\text{D}\nabla C)-\gamma_{c}C, \end{array}$$

where **e**_*z*_ is the outward-pointing unit vector along the ordinate axis, *C* is the concentration of dissolved CO_2_, **u**=(*u*_*x*_,*u*_*z*_) is the fluid velocity and *P* is the fluid pressure. The parameters *K*, *μ*, *ϕ*, *γ*_*c*_ and **g** are, respectively, the medium permeability field, the fluid viscosity, the rock porosity, the reaction rate and acceleration due to gravity. The solute undergoes a first-order reaction and is converted into an inert product with no effect on the solution density, thus the density of the fluid is linearized and takes the form, *ρ*=*ρ*_0_+*β*_*c*_*C*, where *ρ*_0_ and *β*_*c*_ are the density of the pure fluid and the volumetric expansion coefficient. This assumption allows us to use the Boussinesq approximation [32]. The boundary conditions for the above problem are: *C*(*x*,*z*=*H*)=*C*_0_ and *u*_*x*_(0,*z*)=*u*_*x*_(*L*,*z*)=*u*_*z*_(*x*,±*H*)=0, (∂*C*/∂*z*)(*x*,−*H*)=(∂*C*/∂*x*)(0,*z*)=(∂*C*/∂*x*)(*L*,*z*)=0.

The velocity field is represented by using a streamfunction, *Ψ*, formulation, *u*_*x*_=∂*Ψ*/∂*z* and *u*_*z*_=−∂*Ψ*/∂*x*. We can eliminate the pressure field from equation ((2.2)) by satisfying the mass conservation (2.1), resulting in a new set of equations for the unknown field variables (*Ψ*, *C*). For the resulting set of governing equations and boundary conditions we follow the same dimensionless formulation used in Crevillén-García *et al.* [5], where the reader can find a full detailed derivation of formulae and equations. The dimensionless variables and numbers are defined by: *x*′=*x*/*H*, *z*′=*z*/*H*, *Ψ*′=*Ψμ*/*HC*_0_*K*_0_*β*_*c*_*g*, *C*′=*C*/*C*_0_, *t*′=*tC*_0_*K*_0_*β*_*c*_*ρ*/*μϕH*, *β*′_*L*_=*β*_*L*_*C*_0_*K*_0_*β*_*c*_*g*/*D*_0_*μ*, *β*′_*T*_=*β*_*T*_*C*_0_*K*_0_*β*_*c*_*g*/*D*_0_*μ*, *K*′=*K*/*K*_0_, L=L/H, *Ra*=*K*_0_*C*_0_*gβ*_*c*_*H*/*ϕμD*_0_ and *Da*=*γ*_*c*_*μH*/*K*_0_*C*_0_*gβ*_*c*_, where *β*_*T*_ and *β*_*L*_ are, respectively, the longitudinal and transverse dispersion coefficients [37,38]; *K*_0_ and *D*_0_ are reference permeability and diffusion coefficients, respectively; L is the aspect ratio of the domain; *Ra* is the Rayleigh number, related to the buoyancy driven flow; and *Da* is the Damkhöler number, which is the ratio of the chemical reaction rate to the mass transfer rate [32]. In terms of these dimensionless variables and numbers, and dropping the primes for convenience, the following dimensionless governing equations defined in R=[0,L]×[−1,1] stay as:

2.4 $$\frac{\partial }{\partial x}\left(\frac{1}{K}\frac{\partial \Psi }{\partial x}\right)+\frac{\partial }{\partial z}\left(\frac{1}{K}\frac{\partial \Psi }{\partial z}\right)+\frac{\partial C}{\partial x}=0$$

and

2.5 $$\frac{\partial C}{\partial t}-\frac{\partial \Psi }{\partial z}\frac{\partial C}{\partial x}+\frac{\partial \Psi }{\partial x}\frac{\partial C}{\partial z}-\frac{1}{Ra}\left(\frac{\partial J_{x}}{\partial x}+\frac{\partial J_{z}}{\partial z}\right)+DaC=0.$$

The Fickian mass flux **J**=(*J*_*x*_,*J*_*z*_) (Scheidegger-Bear) [38] satisfies **J**=**D**∇*C* and its components are expressed as follows: *J*_*x*_=(1+*β*_*T*_∥∇*Ψ*∥_2_)(∂*C*/∂*x*)+((*β*_*L*_−*β*_*T*_)/∥∇*Ψ*∥_2_)((∂*Ψ*/∂*z*)^2^(∂*C*/∂*x*)−(∂*Ψ*/∂*x*)(∂*Ψ*/∂*z*)(∂*C*/∂*z*)) and *J*_*z*_=(1+*β*_*T*_∥∇*Ψ*∥_2_)(∂*C*/∂*z*)+((*β*_*L*_−*β*_*T*_)/∥∇*Ψ*∥_2_)((∂*Ψ*/∂*x*)^2^(∂*C*/∂*z*)−(∂*Ψ*/∂*x*)(∂*Ψ*/∂*z*)(∂*C*/∂*x*)), where ∥⋅∥_2_ denotes the standard Euclidean norm. Finally, the corresponding dimensionless form of the boundary conditions is: *C*(*x*,1)=1, Ψ(x,±1)=Ψ(0,z)=Ψ(L,z)=0, (∂C/∂z)(x,−1)=(∂C/∂x)(0,z)=(∂C/∂x)(L,z)=0. In this study, we are interested in the long-term behaviour of the system and consequently we will restrict ourselves to the steady-state equations, i.e. by setting ∂*C*/∂*t*=0 in equation (2.5).

### 2.2. Convectively enhanced dissolution numerical simulator

The numerical simulator is built based on an *H*^1^-conforming FEM [39], and the numerical solutions were computed on a shape-regular rectangular partition of $R=[0,\pi /2]\times [-1,1]\subset R^{2}$ comprising 2500 elements (i.e. a computational domain formed by *M*=2601 nodes), employing basis functions of polynomial degree 1. All computations were performed using the AptoFEM finite-element toolkit, documented in Antonietti *et al.* [40], together with the MUMPS linear solver [41,42]. In terms of the CPU time spent by the numerical simulator to compute a single solution of equations (2.4) and (2.5), the choice of different values for the model parameters, *Ra* and *Da*, makes no difference. In this paper, we will restrict ourselves to the case L=π/2, *Ra*=100, *Da*=0.1, *β*_*L*_=*π*/2 and *β*_*T*_=*β*_*L*_/10.

### 2.3. Generation of random permeability fields

Natural media is heterogeneous in a hierarchy of scales, and it is virtually impossible with today’s technologies to resolve this heterogeneity in detail [43]. The permeability values have shown spatial correlation [1–3] and a function that has been extensively used [2,5,7,18,44,45] to represent that correlation is the following squared exponential covariance function:

2.6 $$c({\text{x}}_{i},{\text{x}}_{j})=\sigma^{2}\;\exp\left(\frac{-∥{\text{x}}_{i}-{\text{x}}_{j}{∥}_{2}}{\lambda }\right)\;{\text{x}}_{i},{\text{x}}_{j}\in R,$$

where *λ* represents the correlation length and *σ*^2^ the variance of the process.

The simulator described earlier necessitates the values of the permeability *K* at each of the *M* nodes of the computational domain in order to solve the problem. It is common in groundwater flow models [18] to model *K* as a log-Gaussian random field; this guarantees that *K*>0 in R. In this study, we will model the permeability as log-Gaussian permeability fields and generate samples of the permeability fields at the nodes with the following procedure [46]. Let (Ω,F,P) be a probability space. If we now let Z∼N(m,C), i.e. $Z:\Omega \to R^{M}$ be a multivariate normally distributed random vector with mean and covariance $m={\left(m_{1},\ldots ,m_{M}\right)}^{⊺}=E[Z]\in R^{M}$, $\text{C}=E[(Z-m){\left(Z-m\right)}^{⊺}]\in R^{M\times M}$, respectively, where $m_{i}=E[Z(x_{i})]=m(x_{i})$, *C*_*ij*_=*c*(**x**_*i*_,**x**_*j*_), *i*,*j*=1,…,*M*. Then, given the set of nodes ${\left\{{\text{x}}_{i}\right\}}_{i=1}^{M}$, the vector $Z:={\left(Z(x_{1}),\ldots ,Z(x_{M})\right)}^{⊺}$ is a discrete Gaussian random field. Finally, we set K=exp⁡(Z) to obtain the desired discrete log-Gaussian permeability field, where each of the components of the vector $K\in R^{M}$ corresponds to the value of the permeability at a node of the computational domain.

To generate different samples of Z, we will use the KL decomposition method (see [5,7,46,47]. This method uses an eigen-decomposition of the covariance matrix **C** at the nodes which is then stored for future samples generation. Moreover, the KL expansion may be truncated, which leads to a reduced-dimensional formulation that is critical in the emulator construction. The KL decomposition method is summarized as follows.

The covariance matrix **C** is real-valued and symmetric, and therefore admits an eigen-decomposition [48]: $\text{C}=(\Phi \Lambda^{1/2}){\left(\Phi \Lambda^{1/2}\right)}^{⊺}$, where *Λ* is the *M*×*M* diagonal matrix of ordered decreasing eigenvalues *λ*_1_≥*λ*_2_≥⋯≥*λ*_*M*_≥0, and *Φ* is the *M*×*M* matrix whose columns ***ϕ***_*i*_, *i*=1,…,*M*, are the eigenvectors of *C*. Let $\xi_{i}∼N(0,1)$, *i*=1,…,*M*, be independent and identically distributed (i.i.d.) random variables. We can draw samples from Z∼N(m,C) using the KL decomposition of Z using the following expression [46]:

2.7 $$Z=m+\Phi \Lambda^{\frac{1}{2}}{\left(\xi_{1},\ldots ,\xi_{M}\right)}^{⊺}=m+\sum_{i=1}^{M}\sqrt{\lambda_{i}}\phi_{i}\xi_{i}.$$

The terms $\xi_{i}∼N(0,1)$ are called *KL coefficients*. To be consistent with the non-dimensional formulation of equations (2.4) and (2.5), we generate a set of log-Gaussian permeability fields with point-wise mean 1 by setting m=−(σ/2)I. Let us define the random vector $\xi \in R^{M}$, distributed according to N(0,I). The numerical simulator is considered as a mapping from $\xi \in R^{M}$ to $(C,\Psi )\in R^{M}\times R^{M}$. If we were interested only in one of the two simulator output fields, we could also consider the simulator as either *f*_*c*_:***ξ***↦*C* or *f*_*Ψ*_:***ξ***↦*Ψ*.

In the next section, we will describe how to build a GP emulator.

## 3. Gaussian process emulation of spatial fields in complex simulators

A GP emulator is a statistical approximation of the numerical simulator. In this paper, to build an emulator for a given simulator, we use GP regression methodologies consisting of establishing a prior specification of the functional form of the target simulator which is updated in the light of data provided by using Bayes’ rule, which yields a posterior distribution that can be used for inference. That prior specification consists of providing the model with a mean, a covariance structure and a set of *observed values* (or *targets*) at carefully selected inputs, so-called *design points*. The pair formed by the design points and the observed values at such points is called the *training set*. The mean and covariance functions contain parameters, so-called hyperparameters, that need to be inferred from the training data by solving an optimization problem. For high-dimensional input spaces, the GP model would be impractical. To overcome this optimization issue, we developed an ESGPMR method based on Bayesian inference that is able to recursively find the lowest dimension of the input space for which the GP emulator response surface best approximates the numerical simulator. The method incorporates a continuation routine that helps the optimization algorithm used for the MLE estimates to find adequate initial values for the successive iterations. The continuation routine can be easily made extensive to any existing moderate-dimensional GP emulators that groundwater researchers using commercial GP toolboxes discarded because of the impossibility of estimating the hyperparameters appropriately. In the next section, we only give a brief description of the GP framework, stating our choices for the prior specifications of the GP model, the generation of the training data and the final predictive equations used to approximate the simulator. For a detailed description of conditional distributions and the derivation of the final formulae, we refer the reader to Rasmussen & Williams [21].

### 3.1. Gaussian process emulation framework

In this section, we describe the general GP emulation methodology for scalar functions, to then extend it to vector functions in the next section. Let $g:R^{M}\to R$ be a scalar simulator. The aim of GP emulation is to learn the functional form of the target model *g*(⋅) in the light of a very reduced (due to the time-consuming simulator) set of data. The design points can be regarded as the locations (in the input space) at which we wish to obtain the values of *g*(⋅) to determine the sensitivity of the simulator to different inputs. An exhaustive explanation of the possible choice of design points is addressed in Sacks *et al.* [19]. To generate the set of design points, we simply spread the points to cover the input space, in this case $R^{M}$. There are several methods of sampling the inputs, the most common of which are Latin hypercube sampling (LHS) [49,50] and Sobol sequence sampling [51]. In this paper, guided by the successful results obtained in a previous work, we will use the latter. Given the particular definition of the inputs in our model simulator (ξ∼N(0,I)), one very intuitive way of building a set of *d* design points is to, first, use a Sobol sequence to generate *d* points in [0,1]^*M*^, and second, push the *d* points component-wise through the inverse cumulative distribution function of *M* random variables distributed according to $N(0,\sigma_{d}^{2})$, with $\sigma_{d}^{2}\geq 1$, to, jointly, form the set of design points ${\hat{\xi }}_{j}={\left({\hat{\xi }}_{j}^{1},\ldots ,{\hat{\xi }}_{j}^{M}\right)}^{⊺},\;j=1,\ldots ,d$. Note that by setting *σ*_*d*_>1, we guarantee that the design points are spread enough in $R^{M}$ to cover all the points sampled from the input distribution (N(0,I)), and therefore we will not be missing some key information about the simulator responses at points far from the mean of the input distribution.

Let us denote by *f*(⋅) the GP used to model *g*(⋅). For any $\xi ,\xi^{\prime }\in R^{D}$, for some *D*≤*M*, the GP prior mean function is defined as: m(ξ):=E[f(ξ)] and the covariance function as: $k(\xi ,\xi^{\prime }):=C\text{ov}(f(\xi ),f(\xi^{\prime }))=E[(f(\xi )-m(\xi ))(f(\xi^{\prime })-m(\xi^{\prime }))]$, where E[f(⋅)] and Cov(⋅,⋅) denote the expectation and covariance operators, respectively. One of the methods available in the literature to select the mean and covariance functions for a given model is CV (see [5]). However, the covariance chosen for this GP makes no difference to the scope of the approaches developed in this study, and thus, for simplicity, we will use a mean-zero function and the square exponential (SE) covariance which is given in terms of three hyperparameters as follows [21]:

3.1 $$k(\xi ,\xi^{\prime })=\sigma_{f}^{2}\exp(-\frac{1}{2}{\left(\xi -\xi^{\prime }\right)}^{⊤}\text{diag}(\ell_{1}^{-2},\ldots ,\ell_{D}^{-2})(\xi -\xi^{\prime }))+\sigma_{n}^{2}\delta_{ij},$$

where $\sigma_{f}^{2}$ is the process variance, ℓ=(ℓ_1_,…,ℓ_*D*_) is the length scale, $\sigma_{n}^{2}$ is the noise variance and *δ*_*ij*_ is the Kronecker delta. The hyperparameters are collectively represented by $\theta \;\theta =(\sigma_{f}^{2},\ell ,\sigma_{n}^{2})$. Given the set of design points generated with the method described earlier, ${\hat{\xi }}_{j}={\left({\hat{\xi }}_{j}^{1},\ldots ,{\hat{\xi }}_{j}^{M}\right)}^{⊺}$, *j*=1,…,*d*, we can define the design matrix as $\text{X}=[{\hat{\xi }}_{1},{\hat{\xi }}_{2},\ldots ,{\hat{\xi }}_{d}]$. To avoid numerical instabilities (ill-conditioning of the matrix system), an i.i.d. random noise $\epsilon_{j}∼N(0,\sigma_{n}^{2})$, where $\sigma_{n}^{2}$ is the variance in expression (3.1), is typically introduced into the model, and thus the observed values will take the form $y_{j}=f_{c}({\hat{\xi }}_{j})+\epsilon_{j}$, where *y*_*j*_ is the perturbed simulator output at the design point ${\hat{\xi }}_{j}\in R^{M}$. If we now write $\text{y}={\left[y_{1},\ldots ,y_{d}\right]}^{⊺}$, we can define the *training set* as the pair D={X, y}. Once we have provided the model with a mean-zero function, the SE covariance function (3.1) and the training set D, we can make predictions for new untested inputs $\xi^{*}\in R^{D}$ by using the predictive equations for GP regression [21]:

3.2 $$m_{D}(\xi^{*})=\Sigma (\xi^{*},\text{X}){\left[\Sigma (\text{X},\text{X})+\sigma_{n}^{2}\text{I}\right]}^{-1}\text{y}$$

and

3.3 $$k_{D}(\xi^{*},\xi^{*})=k(\xi^{*},\xi^{*})-\Sigma {\left(\xi^{*},\text{X}\right)}^{⊺}{\left[\Sigma (\text{X},\text{X})+\sigma_{n}^{2}\text{I}\right]}^{-1}\Sigma (\xi^{*},\text{X}),$$

where the (*i*,*j*)th entry of $\Sigma (X,X)\in R^{d\times d}$ is given by $k({\hat{\xi }}_{i},{\hat{\xi }}_{j})$ and $\Sigma (\xi^{*},X)={\left(k(\xi^{*},{\hat{\xi }}_{1}),\ldots ,k(\xi^{*},{\hat{\xi }}_{d})\right)}^{⊺}$. Expression (3.2) for the GP posterior mean $m_{D}$ can be then used to emulate the simulator output at any new input ξ*, i.e. we can write $f(\xi^{*}):=m_{D}(\xi^{*})\approx g(\xi^{*})$. Expression (3.3) provides the predictive variance in the estimation of the output.

### 3.2. Reduced-rank approximation of the output space

In this section, we use the method proposed by Higdon *et al.* [26]. The idea is to use PCA to project the original simulator outputs onto a lower-dimensional space spanned by an orthogonal basis. This is done via singular value decomposition (SVD) as we detail later. Once in the PCA framework, the outputs can be expressed as a linear combination of PCA basis vectors (or the principal components (PCs)) with coefficients treated as independent univariate GPs with distinct sets of correlation lengths. This allows us to build separate GP emulators (as many as PCs considered) to estimate the coefficients of new outputs at untested inputs in the PCA basis. Then we use a linear map for reconstruction to the original output space. By using orthogonal projection, we guarantees a minimal average reconstruction error. The error considered for comparisons between two vectors throughout this paper will be the *L*^2^-norm relative error (RE) unless stated otherwise. For the two vectors **x**=(*x*_1_,…,*x*_*M*_) and **y**=(*y*_1_,…,*y*_*M*_), we define the *L*^2^-norm RE between **x** and **y** as

3.4 $$\text{RE}(\text{x},\text{y})=\frac{∥\text{x}-\text{y}{∥}_{2}}{∥\text{x}{∥}_{2}},$$

where ∥**x**∥_2_ is the Euclidean norm.

Let us consider a simulator (e.g. *f*_*c*_) which receives inputs in $R^{M}$ and returns outputs in $R^{M}$ (instead of R). Then the GP emulator described in §(a) would not work. Let Y be the *M*×*d* matrix with column *j* the *j*th run of the simulator. The dimension reduction in the output space can be described as follows:

(i) Subtract off the mean for each dimension *M* to obtain the centred version of the matrix Y, $Y^{\prime }.$
(ii) Multiply the centred matrix $Y^{\prime }$ by the normalization constant $1/\sqrt{d-1}$ to obtain $Y^{\prime \prime }$.
(iii) Compute the SVD of $Y^{\prime \prime }$ and obtain the *M*×*M* matrix ***U*** whose columns $u_{j},\;j=1,\ldots ,M$, are the PCs of the PCA basis.
(iv) Project the original centred data into the orthonormal space to obtain the matrix of coefficients, ***α***=(*α*_*ij*_), *i*=1,…,*M*, *j*=1,…,*d*.
  An orthonormal basis for a lower-dimensional space of dimension *r*<*M* is given by the first *r* PCs of ${\left\{u_{j}\right\}}_{j=1}^{M}$. Thus a *reduced-rank approximation* of $Y^{\prime \prime }$, ${\tilde{Y}}^{\prime \prime }$ can be obtained by using the first *r* columns of ***U*** and the first *r* rows of ***α***.

Now we can build *r* separate and independent GPs from the input space $R^{M}$ to R as described in §(a) by generating also *r* separate training sets. In this case, the design points in all the training sets are the same, $\text{X}={\left\{{\hat{\xi }}_{j}\right\}}_{j=1}^{d}$, with ${\hat{\xi }}_{j}\in R^{M}$, and the set of observed values are the coefficients of the PCs computed above, i.e. the first *r* rows of ***α***. Thus, for any untested input $\xi^{*}\in R^{M}$, we use expression (3.2) for each of the *r* GPs to estimate the *r* coefficients. These are then stored in vector form and can be mapped back to the original output space to obtain the final GP prediction ${\text{y}}^{*}\in R^{M}$. We can test the accuracy in the prediction by running the numerical simulator at the same input ξ* and compare the result ${\text{y}}_{\mathrm{true}}\in R^{M}$ with y*. Unfortunately for high-dimensional input spaces this approach is not valid and an additional input space dimension reduction must be performed.

In the next section, we propose a method for overcoming the limitation that GPs have in high-dimensional input spaces. This methodology is then combined with the output space reduction method above to build a GP emulator for the full simulator.

### 3.3. The empirical simultaneous Gaussian process model reduction method

**Figure 1.**
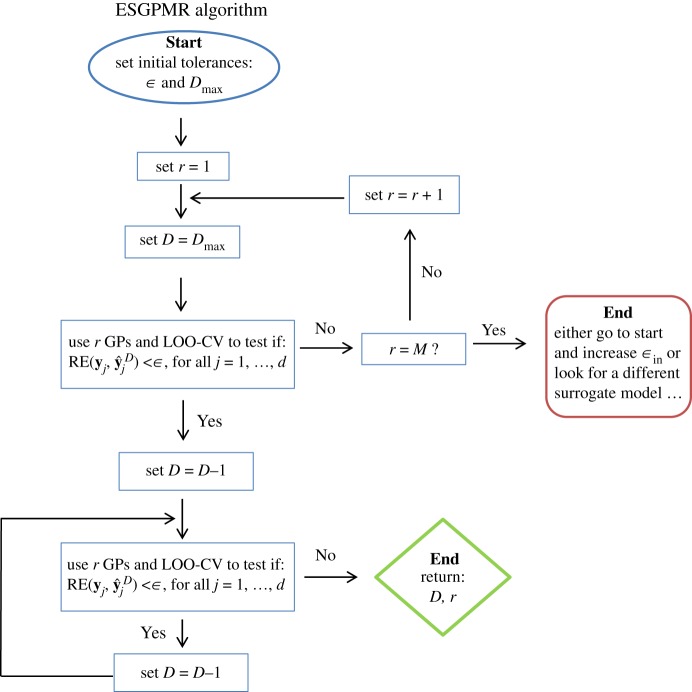
ESGPMR algorithm for code implementation.

Let us clarify the notation first. Suppose we have a set of *d* design points ${\hat{\xi }}_{j}\;\in R^{M}$ generated as described in §(a). We run the simulator at those points to obtain the corresponding *true* *d* output fields **y**_1_,…,**y**_*d*_ where the fields **y**_*j*_ are reshaped to form the columns of the *M*×*d* outputs matrix Y. Then we use the dimension-reduction method described in §(b) to obtain the PCA basis and the matrix of coefficients ***α***=(*α*_*ij*_), *i*=1,…,*M*, *j*=1,…,*d*. We denote by ${\tilde{Y}}^{r}$ the reduced-rank approximation of Y obtained by considering the first *r*≤*M* PCs of the PCA basis whose columns ${\tilde{\text{y}}}_{j}^{r},\;j=1,\ldots ,d$ are the correspondent reduced-rank approximations of the observed fields **y**_*j*_, *j*=1,…,*d*. As we wish to reduce the dimension *M* of the original input space, let us define, for any *D*≤*M*, the training sets as: ${\left\{D_{i}^{D}=(X^{D},\alpha_{i})\right\}}_{i=1}^{r}$, where $X^{D}=[{\hat{\xi }}_{1}^{D},\ldots ,{\hat{\xi }}_{d}^{D}]$ is the truncated design matrix with *D* denoting the first *D* components used from the whole set of *M* (e.g. for ${\hat{\xi }}_{1}={\left(\xi_{1}^{1},\ldots ,\xi_{1}^{D},\ldots ,\xi_{1}^{M}\right)}^{⊺}$ we have ${\hat{\xi }}_{1}^{D}={\left(\xi_{1}^{1},\ldots ,\xi_{1}^{D}\right)}^{⊺}$), and ***α***_*i*_=(*α*_*ij*_), *j*=1,…,*d*. The ESGPMR algorithm (figure 1) can finally be described as follows:

(i) Set accuracy tolerance *ε* and maximum dimension of the input space to be considered $D_{\max}$.
(ii) Set *r*=1.
(iii) Find a reduced-rank approximation ${\tilde{Y}}^{r}$ of the original Y by using the first *r* PCs.
(iv) Set $D=D_{\max}$.
(v) Form the training sets ${\left\{D_{i}^{D}\right\}}_{i=1}^{r}$ and build *r* independent GPs (as described in §(b)). Follow the leave-one-out cross-validation (LOO-CV) approach and use the previous GPs to predict the fields at the leave-out points ${\hat{\xi }}_{j}^{D},\;j=1,\ldots ,d$, and then check if the following expression holds:
  3.5 $$\text{RE}({\text{y}}_{j},{\hat{y}}_{j}^{D})<\varepsilon ,\;\forall j=1,\ldots ,d,$$
  where **y**_*j*_ are the columns of Y (the true fields) and ${\hat{y}}_{j}^{D}$ denotes the predicted field at point ${\hat{\xi }}_{j}^{D}$.
  If expression (3.5) does not hold, set *r*=*r*+1 and go to (iii) (to refine the reduced-rank approximation error). If expression (3.5) holds, set $D=D_{\max}-1$ and go to (v) (to reduce the dimension of the input space) until the expression does not hold, and then return *D* and *r*.

Note that the choices of *ε* and $D_{\max}$ are problem-dependent and completely heuristic. In the next section, we will discuss how to choose values for those tolerances by examining the data, although this ultimately depends on the end-user criteria.

### 3.4. Leave-one-out cross-validation and hyperparameters estimation

Estimates of the unknown hyperparameters $\theta \;\theta =(\sigma_{f}^{2},\ell ,\sigma_{n}^{2})$ in expression (3.1) need to be inferred from the data provided to the model. This step is a crucial part of GP emulation. While this is quite simple to solve in one-dimensional problems, it really becomes an optimization issue when dimension increases. In this section, we describe how to estimate those parameters from the training set even if the dimension of the input space is large. To estimate the hyperparameters, we use a technique known as leave-one-out cross-validation (LOO-CV) (see [5,21]). LOO-CV consists of using all training set data but one point (the *leave-out*) for training, and computing the model prediction error on the leave-out point. This process is repeated until all available *d* points have been exhausted. We use each of the *d* leave-out training sets and a conjugate gradient optimizer to obtain estimates of the hyperparameters by maximizing the log marginal likelihood (3.6) with respect to the hyperparameters:

3.6 $$\log p(\text{y}|\text{X},\theta \;\theta )=-\frac{1}{2}{\text{y}}^{⊺}{\left(\Sigma +\sigma_{n}^{2}\text{I}\right)}^{-1}\text{y}-\frac{1}{2}\log|\Sigma +\sigma_{n}^{2}\text{I}|-\frac{n}{2}\log2\pi .$$

**Figure 2.**
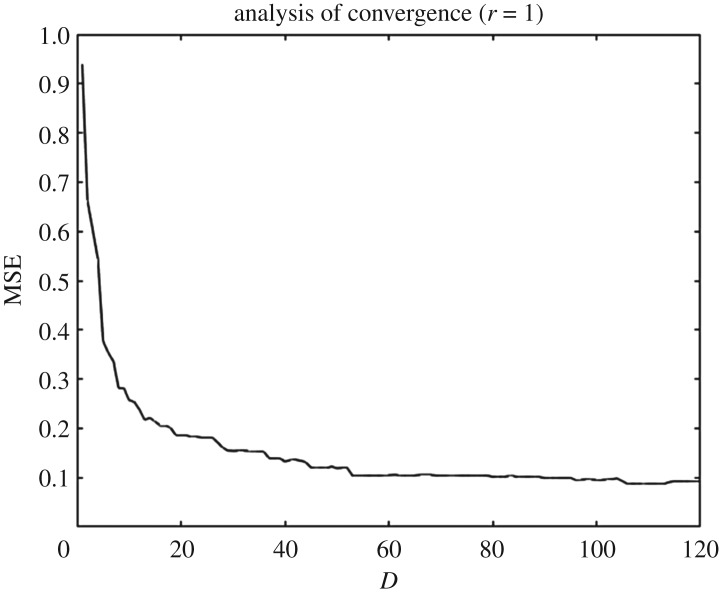
MSE against the number of KL coefficients or input space dimension *D*. These data correspond to the emulation of the first PC component.

The prediction errors during the LOO-CV scheme are quantified through the mean square error (MSE):

3.7 $$\text{MSE}=\frac{1}{d}\sum_{j=1}^{d}{\left(y_{j}-m_{j}\right)}^{2},$$

where *m*_*j*_ is the predicted expected value given by expression (3.2) and *y*_*j*_ is the corresponding observed value both at the same (leave-out) input ${\hat{\xi }}_{j}$. Note that the MSE depends only on the mean predictions, and thus, sometimes, different CV measures which also take into account predictive variances, such as the negative log validation density loss [21] or the Dawid score [52], might be preferred. For the purpose of this study, the MSE gives us the relevant information about the LOO-CV predictions we need for assessment. For an optimal performance of the optimization algorithm and for avoiding failure due to the existence of possible marginal likelihood multiple optima, a continuation routine must be included in all the independent GP emulators described earlier. This is straightforward and can be implemented as follows:

(i) Consider the training sets as in §(c), i.e. for any *D*≤*M*: ${\left\{D_{i}^{D}=(X^{D},\alpha_{i})\right\}}_{i=1}^{r}$. Without loss of generality, let us set *r*=1. The method is exactly the same for the other *r*−1 GPs.
(ii) Estimate the hyperparameters by finding the MLE of expression (3.6) for the *D* one-dimensional problems until a maximum value $D_{\max}$ (large), i.e. by using the *D*th KL coefficient of each of the *d* design points in the training set. We obtain $\theta^{\left(\text{ini}\right)}={\left(\sigma_{f}^{\left(\text{ini}\right)},l_{1}^{\left(\text{ini}\right)},\ldots ,l_{D}^{\left(\text{ini}\right)},\sigma_{n}^{\left(\text{ini}\right)}\right)}^{⊺}$. Note that the importance here lies in the length scales arising from the anisotropic covariance function. Note also that we do not need to compute these values *a priori* and we can do each calculation just before each iteration as needed (depending on $D_{\max}$).
(iii) Start the iteration over the number of KL coefficients *D*. For *D*=1 perform a LOO-CV scheme by using the values obtained in (ii) as initial guess for the estimation of hyperparameters to be used in the GP. Store the hyperparameters as $\theta \;\theta^{\left(1\right)}={\left(\sigma_{f}^{\left(1\right)},\ell_{1}^{\left(1\right)},\sigma_{n}^{\left(1\right)}\right)}^{⊺}$.
(iv) Repeat the iterations until $D=D_{\max}$, and for *D*>2 take as initial guess the previous estimation of the hyperparameters in the lower-dimensional space and the first estimation obtained in (ii) for the next component. For example, for *θ*^(2)^ take as initial guess: ${\left(\sigma_{f}^{\left(1\right)},\ell_{1}^{\left(1\right)},\ell_{2}^{\left(\mathrm{ini}\right)},\sigma_{n}^{\left(1\right)}\right)}^{⊺}$.
  Store the MSE for all the *D* iterations to examine convergence. By inspecting figure 2, we can estimate a value for $D_{\max}$ and refine the model.

## 4. Numerical results

In this section, we discuss the results obtained by applying the reduction and GP emulation methods to the model problem introduced in §2. Let us consider the numerical simulators *f*_*c*_:***ξ***↦*C* and *f*_*Ψ*_:***ξ***↦*Ψ* with $\xi \in R^{M}$, distributed according to N(0,I). $C\in R^{M}$ and $\Psi \in R^{M}$ are the concentration and streamfunction, respectively. Let us show how we build the GP emulator for the concentration simulator *f*_*c*_. Exactly the same procedure applies to *f*_*Ψ*_. The first step is to build a training set. We generate *d*=256 points as described in §(a), i.e. we use a Sobol sequence to generate *d* points in [0,1]^*M*^, and, second, push the *d* points component wise through the inverse cumulative distribution function of *M* random variables distributed according to $N(0,\sigma_{d}^{2})$, with *σ*_*d*_=1.32, to, jointly, form the set of design points ${\hat{\xi }}_{j},\;j=1,\ldots ,d$. For the choice of *σ*_*d*_, we tested the GP simulator for three different values: *σ*_*d*_=1.32 was the one providing more accuracy in the LOO-CV test. To exploit the properties of Sobol sequences and spread the points in the space in an optimal manner, it is recommended [53] that the generated samples are a power of 2. In this study, we use *d*=2^8^=256. A lower number of design points might be used with the same degree of accuracy in the results (see [5]), although as our decisions for the model specifications are based mainly in the LOO-CV technique, we need a relatively large amount of experimental data. For those design points we run the simulator and obtain the correspondent concentration fields $f_{c}({\hat{\xi }}_{1})=C_{1},\ldots ,f_{c}({\hat{\xi }}_{d})=C_{d}$.

The key parameters used to characterize the heterogeneity of the porous medium appeared in expression (2.6). A detailed analysis of the impact of heterogeneity on the concentration profiles and the streamfunction fields has been conducted previously [5,24]; in these earlier works, a measure of the amount of CO_2_ adsorbed through the top boundary in a process of CO_2_ storage is computed from both the heterogeneous case and the one for an equivalent homogeneous medium characterized by a constant permeability equal to the mean permeability in the domain. For our simulations, the value of *λ* will be set to *λ*=0.5. This value has been taken from the ranges suggested in the literature (see [18]). The existence of bifurcating branches of solutions in the C-ED model (see [32]), i.e. there is not always guarantee of a unique observed value at a single design point, might lead to inaccurate training data if large values of *σ*^2^ are considered and no classification techniques are employed [5]. Thus, for simplicity and without loss of generality, we will set *σ*^2^=0.1. Note that the choice of *σ*^2^ does not directly affect the applicability of the ESGPMR method proposed in this paper but the uniqueness of the simulator outputs. Consequently, the use of larger values for *σ*^2^ would probably necessitate using additional pre-processing tools to classify the variety of branches of observed data before forming the training set. This scenario has been treated in detail previously [5]. Note that for models where there is a one-to-one correspondence between inputs and outputs, the ESGPMR can be applied without restriction.

**Figure 3.**
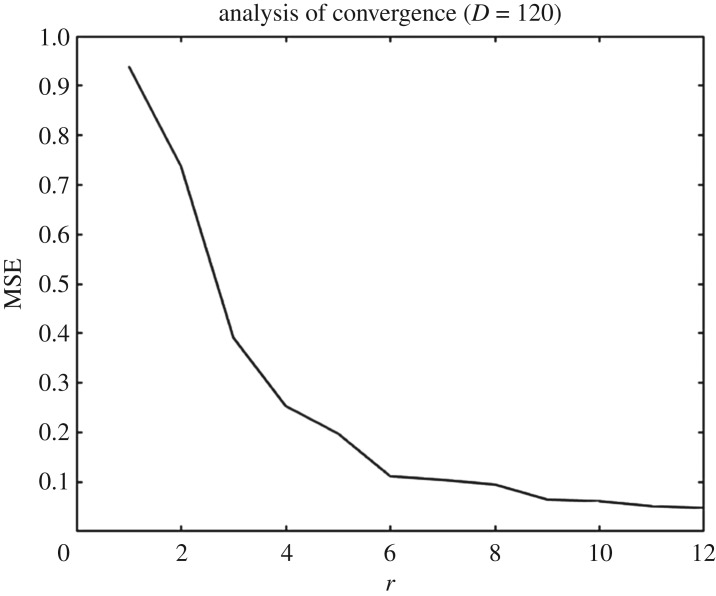
MSE against the number of PCs or output space dimension *r*. These data correspond to a GP emulation with *D*=120.

**Figure 4.**
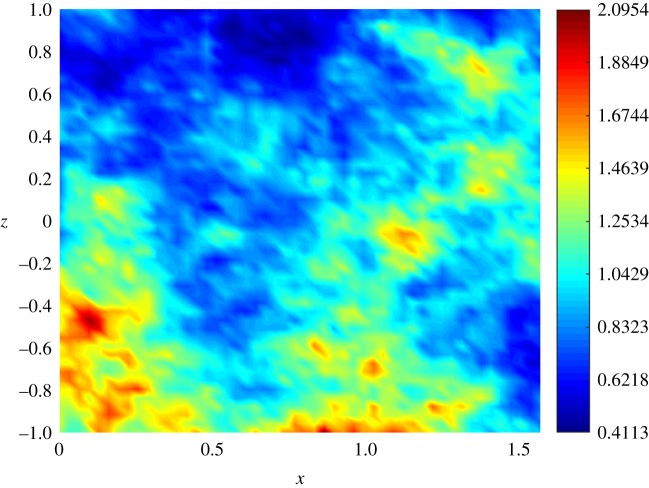
Permeability field used for the prediction of the concentration and streamfunction fields shown in figures 5 and 6.

**Figure 5.**
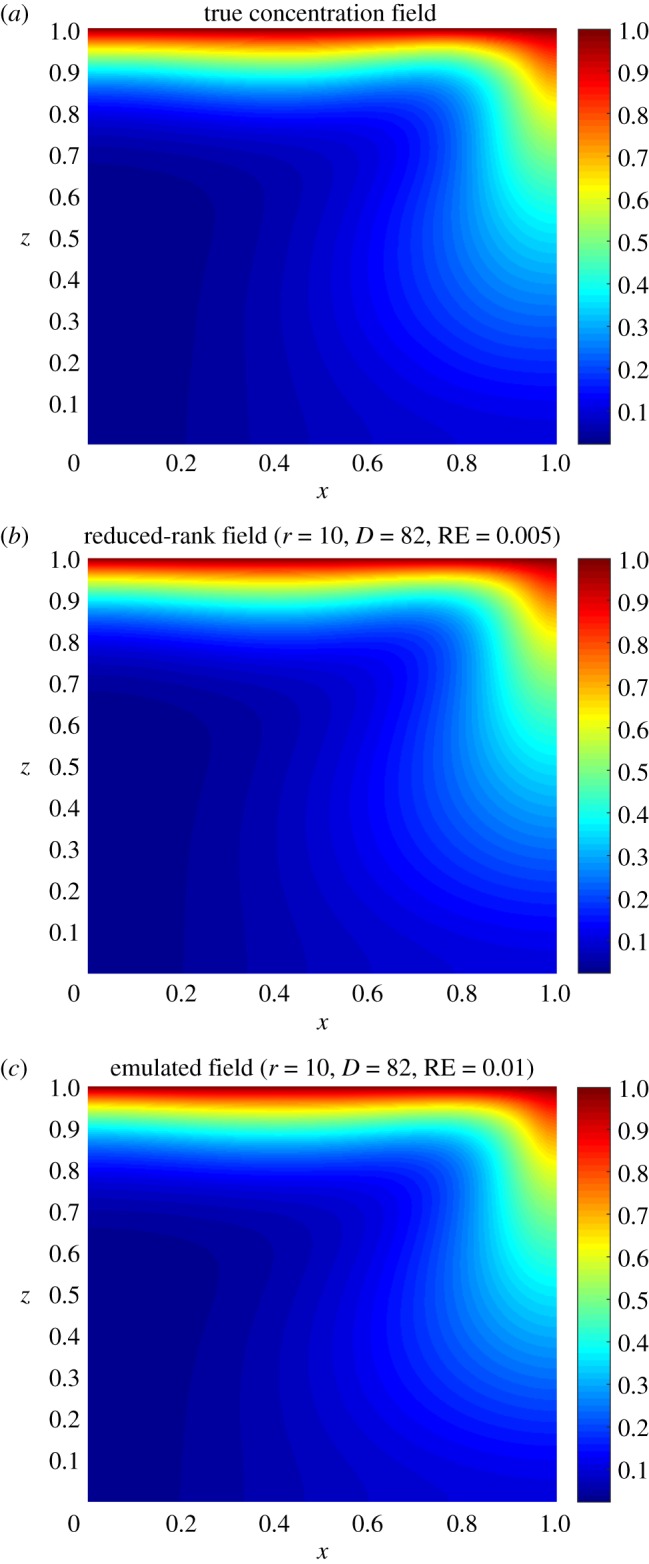
True (*a*), reduced rank (*b*) and predicted (*c*) concentration fields for the permeability shown in figure 4. The dimension of the input (*D*) and output (*r*) spaces and the relative error (RE) achieved are also reported.

**Figure 6.**
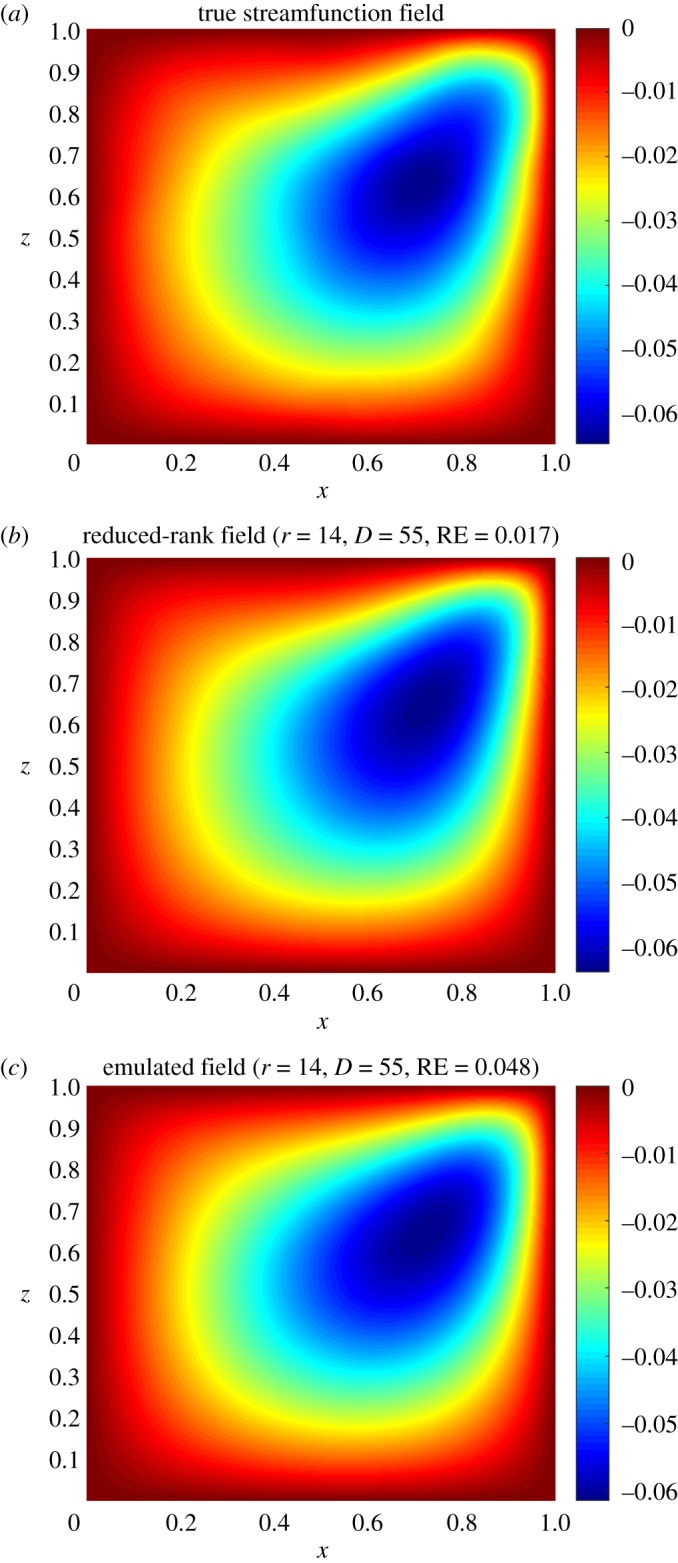
True (*a*), reduced rank (*b*) and predicted (*c*) streamfunction fields for the permeability shown in figure 4. The dimension of the input (*D*) and output (*r*) spaces and the relative error (RE) achieved are also reported.

Once we have generated the training set and decided the prior specifications for the GPs, we use the ESGPMR algorithm to reduce the dimensionality of the input and output spaces in order to bring the original model to a more computationally tractable and accurate problem. Tables 1 and 2 show the results for different accuracy tolerances *ε* obtained from the set of concentration and streamfunction fields, respectively. It also shows the number of KL coefficients used for the input space, the number of PCs from the PCA basis for the output space and the overall relative (maximum) error achieved. We observe that (as one might expect) the more dimensions that are considered, the more accurate is the overall approximation of the GP emulator. This is also a sign that the ESGPMR algorithm is well designed. To set an optimal value for $D_{\max}$, we need to conduct a first experiment allowing $D_{\max}$ to be large enough to allow us to investigate, for instance by visual inspection, some signs or numerical evidence of convergence. Figure 2 provides us with a valuable information about the latter. For this model, a sensible value for $D_{\max}$ might be 100 (higher or lower values are at user discretion). Note that this limit is only illustrative as figure 2 is only considering the first GP emulator or *r*=1. Although the value of $D_{\max}$ is just a reference, using more DoF does not seem to be sensible as it could lead to additional numerical errors as well as an exponential increase of the computational cost. It is important to note that $D_{\max}$ is just a user’s auto-imposed limit depending on the user’s own computational resources, and therefore it is related to dimension reduction, while *ε* is related to the accuracy of the GP results. Thus, the choices of $D_{\max}$ and *ε* are made independently. In these terms, if the user has not reached the desired accuracy in the GP predictions for either a tolerance *ε* or a relatively large value of $D_{\max}$, it can be concluded that GP emulation is not *a priori* (one can always try with different prior mean, covariance or likelihood functions) a recommendable surrogate model for the numerical simulator. Figure 3 shows numerical evidence of how the reduction of the MSE depends on the number of PCs considered. Figure 4 shows the permeability field used to compute the concentration and the streamfunction outputs shown in figures 5 and 6. Figure 5 shows the results obtained by using the GP emulator with *D*=82 and *r*=10 to predict the concentration output field at one untested point $\xi^{*}\in R^{M}$. The RE between the true and the reduced rank approximation was 0.005. The RE between the true and the predicted was 0.01. Figure 6 shows the results for the same input considered in figure 5, where in this case the best resolution achieved was using *D*=55 and *r*=14. We highlight here that different values of *D* and *r* are needed for each GP emulator to achieve the desired tolerance, i.e. while for the concentration we needed *D*=5 and *r*=3 to achieve a tolerance of 0.05, for the streamfunction we needed *D*=55 and *r*=14. Furthermore, both algorithms were unable to refine further, and thus the lowest tolerances we could achieve in this study were *ε*=0.01 for the concentration and *ε*=0.05 for the streamfunction.

**Table 1.** Relative errors between the true and reduced-rank approximation RE_*true*-*red*_ and between and the true and the predicted concentration fields RE_*true*-*pred*_ for three different tolerances *ε*. The numbers of PCs (PC) and KL coefficients (KL) used are also provided.

*ε*	PC	KL	RE_*true*-*red*_	RE_*true*-*pred*_
0.050	3	5	0.034	0.047
0.025	6	14	0.017	0.023
0.010	10	82	0.005	0.010

**Table 2.** Relative errors between the true and reduced-rank approximation RE_*true*-*red*_ and between and the true and the predicted streamfunction fields RE_*true*-*pred*_ for three different tolerances *ε*. The numbers of PCs (PC) and KL coefficients (KL) used are also provided.

*ε*	PC	KL	RE_*true*-*red*_	RE_*true*-*pred*_
0.100	5	6	0.069	0.095
0.075	10	18	0.036	0.072
0.050	14	55	0.017	0.048

**Figure 7.**
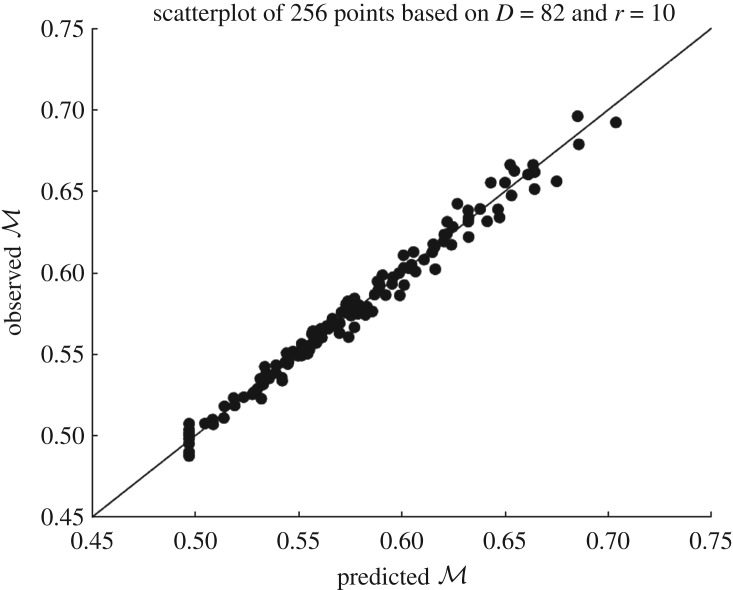
Scatterplot of the mass 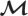 computed from the 256 observed concentration fields in the training set and the predicted mass obtained from the emulated concentration fields at the same design points. The line *y*=*x* is used as the reference for the best possible performance.

Before finishing this section, let us see another application. We can use the GP emulator to approximate scalar-valued quantities of interest from any of the output fields, for instance we can compute the total mass of dissolved solute in the domain R given by BarbaRossa *et al*. [34]: $M=\int_{R}C$. In this case, we only need to emulate the concentration field *C* for the new input and then compute the integral over R. An intuitive and qualitative way of measuring how close our GP predictions are to the observed values is through *scatterplots*. This consists of plotting the pairs (predicted outputs, observed outputs) along with the line *y*=*x* and checking that the scattered points are not ‘far away’ from the straight line. For this application, we used the data stored during the LOO-CV scheme for the GP above and computed the set $M_{1}^{*},\ldots ,M_{d}^{*}$ from the emulated concentration fields. Then we computed $M_{1},\ldots ,M_{d}$ directly from the simulator concentration outputs *C*_1_,…,*C*_*d*_. Figure 7 shows the scatterplot for the observed values (*Y*-axis) against the predicted values (*X*-axis).

In this study, the GP emulation was implemented using the GPML MATLAB Toolbox v.3.4 [21].

## 5. Conclusion

In this paper, we developed a methodology based on dimensionality reduction and GP emulation for surrogate modelling in SPDEs. The technique can be applied without modification to any model involving vector-valued functions and vector-valued inputs. The ESGPMR algorithm was able to simplify the original mathematical problem while retaining the accuracy in the results. In particular, for the emulation of concentration fields, one of the GP emulators was able to reduce the dimensionality of the input and output spaces from *M*=2601 to *D*=112 and from *M*=2601 to *r*=20, respectively, for an overall tolerance of *ε*=0.01.

The ESGPMR algorithm provides the end-user with a tool for assessing if GP emulation is an efficient surrogate model for a given computationally expensive numerical simulator. This applies to either numerical models where it is not feasible to meet the desired resolution in the GP predictions or when the original problem cannot be adequately reduced to a more tractable model (i.e. $D_{\max}$ and *r* are found to be extremely large for the tolerances given).

## Data accessibility

Our data are available in Dryad at https://doi.org/10.5061/dryad.3g280 [54].
